# Physician burnout: prevention strategies

**DOI:** 10.47626/1679-4435-2021-713

**Published:** 2021-12-30

**Authors:** Paulo Guen-iti Matsuzaki, Fernando Akio Mariya, Leandro Issamu Ueno, Maria José Fernandes Gimenes

**Affiliations:** Medicina do Trabalho, Faculdade de Medicina do ABC, Santo André, SP, Brazil.

**Keywords:** burnout, prevention, physicians, burnout, prevenção, médicos

## Abstract

Burnout syndrome is a response to occupational stress that consists of emotional exhaustion, depersonalization, and reduced personal fulfillment. It may affect health care professionals, requiring due attention and the development of preventive mechanisms. The objective of this study was to identify possible ways to prevent the onset of burnout among physicians. A literature review was conducted in PubMed and SciELO databases. The search resulted in 16 articles on the subject, of which 11 conducted individual-focused interventions, four focused on the work environment, and one focused on both aspects. In conclusion, reducing burnout levels may benefit both physicians and patients, and conducting approaches focused on both the individual and the work environment is essential. However, further research on physician burnout prevention is needed.

## Resumo

A síndrome de *burnout* é uma resposta aos estressores decorrentes do trabalho e é caracterizada por esgotamento emocional, despersonalização e redução da realização pessoal. Essa síndrome pode afetar os profissionais de saúde, merecendo atenção e o desenvolvimento de mecanismos preventivos. O objetivo deste estudo foi identificar possíveis formas de prevenção da síndrome de *burnout* em médicos. Foi realizada uma revisão bibliográfica, com buscas nas bases de dados PubMed e SciELO. Foram identificados 16 artigos sobre a temática, dos quais 11 promoviam intervenções preventivas focadas no indivíduo, quatro focavam no ambiente de trabalho e um abordava a prevenção com foco no indivíduo e no ambiente de trabalho. Conclui-se que a redução dos índices de *burnout* pode beneficiar tanto os médicos quanto os pacientes e que a abordagem preventiva com base no indivíduo e no ambiente de trabalho são essenciais. Entretanto, é necessário que sejam realizadas mais estudos sobre a prevenção da síndrome de *burnout* em médicos.

## INTRODUCTION

### OVERVIEW

The term “burnout syndrome” was first used in 1974 by Freudenberger when he noticed a decrease in enthusiasm in his work compared to previous times. He correlated the lack of stimulus originated from the lack of emotional energy with the feeling of burnout. In 1981, Maslach & Jackson described burnout syndrome as a work-related impairment of mental health that consists of emotional exhaustion, individual depersonalization and reduced personal fulfillment. Emotional exhaustion is a state in which all emotional resources are exhausted; depersonalization refers to a distant, skeptical, and negative approach to the people under one’s care; and reduced personal fulfillment is related to feelings of inefficiency and negative feelings about oneself.^1^

Burnout syndrome often affects those with overwhelming, high-demand jobs and those who work directly with other people, such as teachers and health care professionals. Moreover, some studies suggest a high prevalence of burnout syndrome among physicians and that 1/3 of physicians are considerably affected by burnout at some point in their careers.^2^ Burnout syndrome among physicians has been associated with an increase in medical errors, lower patient satisfaction, prolonged recovery periods for physicians, and lower job satisfaction.^3^

Within this context, burnout syndrome is a health issue that may result in absence from work and sick leave, consequently leading to company expenses, complications related to employee absence, and reduced service quality and productivity.^3^

### RISK FACTORS

According to its definition, burnout syndrome is caused by occupational exposure to stress and has a multifactorial etiology. According to Gil-Monte & Peiró (apud Moreira et al.^1^), possible risk factors for physician burnout may be classified as facilitators or triggers. Facilitators are person-related situations that may function as predictors or inhibitors of the effect stress can have on that person (resilience, job satisfaction, fatigue, and anxiety, among others), whereas triggers are factors related to the workplace (relationship with co-workers, lack of or insufficient material to do your job properly, and demand for results, among others).^1^

Loss of autonomy regarding how much time to spend with patients, treating the data and not the patient, too many rules limiting the time physicians can spend with a patient, insufficient pay, and the sense of powerlessness due to the lack of resources to treat patients properly are among possible causes of physician burnout.^4^

### PREVENTION

Burnout prevention programs may be individual-focused, focused on the organization of the work environment, or a combination of both. Individual-focused programs usually consist of behavioral measures aimed at coping with occupational issues through social support or different kinds of relaxation exercises.

Programs focused on the work environment, on the other hand, include changes in work procedures, task restructuring, and work evaluation and supervision aimed at decreasing job demand and increasing job control and the level of participation in decision-making.^5^

Both individual- and workplace-focused interventions may reduce burnout rates. Better outcomes are believed to result from conducting both interventions simultaneously rather than separately.^6^

### OBJECTIVE

To identify possible interventions to prevent physician burnout.

## METHODS

A literature review was conducted in PubMed and SciELO databases for articles published in the last 11 years (2009-2019). The following keywords were used: physician, burnout, prevention, *prevenção*, and *médicos*. The search retrieved 544 articles in PubMed and 35 in SciELO.

After screening of titles and abstracts, 16 articles were included in this study according to the following criteria: articles published in the last 11 years (from 2009 to 2019); in Portuguese or English; conducted with physicians; and focused on prevention methods and risk factors for burnout syndrome. Exclusion criteria were studies conducted with health professionals other than physicians, with medical students or students from other fields, and which only assessed protective and risk factors without focusing on prevention.

## RESULTS

**Figure 1:**
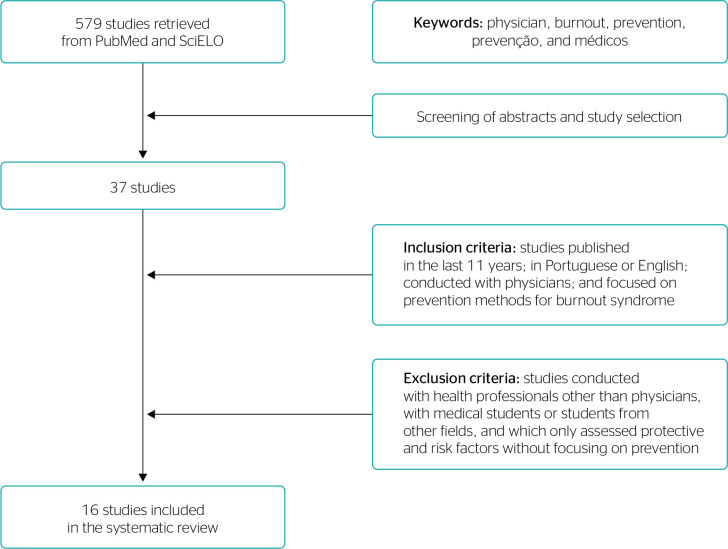
Flow diagram of study selection.

The search in the databases found 579 articles. After reading titles and abstracts, 37 articles were selected for full reading. Finally, the selection of articles according to the exclusion criteria resulted in the inclusion of 16 studies in this literature review (Figure 1).^7-22^

Study characteristics, interventions, and follow-up results are presented in Table 1.

**Table 1:** Study characteristics, interventions, and follow-up results (in alphabetical order per author)

Study	Country	No. of participants	Intervention	Control group	Study duration	Burnout assessment tool	Results at 6 months of follow-up	Results at 1 year of follow-up
Ali et al.^7^	USA	45 physicians	Interrupted on-call schedule	Continuous on-call schedule	9 months	National Study of the Changing Workforce	Decreased burnout levels	-
Amutio et al.^8^	Spain	42 physicians	MBSR	None	1 month	MBI	Decreased burnout levels	Decreased burnout levels
Asuero et al.^9^	Spain	68 physicians	Mindfulness-based coping strategies Mindfulness practice Yoga Group discussion	Wait list	8 weeks	MBI	Decreased burnout levels	-
Bragard et al.^10^	Belgium	96 physicians	30-Hour training on communication skills 10-Hour training on stress management in small groups (≤ 7 participants)	Wait list	2 months	MBI	Decreased burnout levels	-
Goldhagen et al.^11^	USA	30 residents	Two or three 3-hour sessions of mindfulness-based activities	-	8 weeks	Oldenburg Burnout Inventory	Decreased burnout levels	-
Gunasingam et al.^12^	Australia	31 physicians	Four debriefing sessions	None	2 months	MBI	Same burnout levels	-
Krasner et al.^13^	USA	70 physicians	Continuing medical education	-	12 months	MBI	Decreased burnout levels	-
Linzer et al.^14^	USA	135 physicians	Improvement of team communication Changes in the workplace Quality improvement projects targeting physicians’ concerns	None	18 months	MBI	-	Decreased burnout levels
Lucas et al.^15^	USA	62 physicians	Random sequences of 2-week rotations	Random sequences of 4-week rotations	1 month	MBI	Decreased burnout levels	-
Martins et al.^16^	Brazil	74 residents	Self-care workshops	None	2 months	MBI	Same burnout levels	-
Milsten et al.^17^	USA	15 residents	Self-administered psychotherapeutic tools	None	3 months	MBI	Same burnout levels	-
Parshuram et al.^18^	Canada	47 residents	12-Hour shift	16- and 24-hour shifts	6 months	MBI	Same burnout levels	-
Ripp et al.^19^	USA	188 residents	Changes in residency working hours, breaks, and an admission system in 2011	Data from 2008-2009	1 year	MBI	Same burnout levels	-
Verweij et al.^20^	Norway	65 physicians	MBSR	Wait list	8 weeks	MBI	Decreased burnout levels	-
Weight et al.^21^	USA	628 residents	12-Week exercise programs	None	12 weeks	MBI	Decreased burnout levels	-
West et al.^22^	USA	74 physicians	Group discussion with mindfulness-related elements	None	9 months	MBI	Decreased burnout levels	Same results as the 3-month evaluation

MBI = Maslach Burnout Inventory; MBSR = mindfulness-based stress reduction program.

Intervention durations ranged from 2 to 18 months. Burnout prevention strategies may be focused on organizational changes or individual aspects. Of the selected studies, five were focused on the organization of the workplace,^7,14,15,18,19^ 11 were individual-focused,^8,9,11-13,16,17,20-22^ and only one was focused on both interventions concurrently.^10^

Fourteen studies were clinical trials consisting of intervention and control groups. Control groups did not receive any intervention in seven studies,^8,13,15,17,18,21,22^ and were composed of individuals on the wait list in three studies.^9,10,20^ The shift schedule differed between the control and intervention groups in three studies.^7,15,18^ One study compared the control group with results obtained from a previous study.^19^

Burnout assessment instruments varied between studies. The Maslach Burnout Inventory (MBI) was the most used, accounting for 14 studies.^8-10,12-22^ The Oldenburg Burnout Inventory^11^ and the National Study of the Workforce^7^ were used in only one study each.

Regarding the follow-up period, 15 studies conducted follow-up immediately or up to 6 months after the intervention.^7-22^ During the follow-up period, eight studies identified a decrease in burnout rates.^7-9,13,15,20-22^ On the other hand, no study conducted assessments or follow-up between 6 months and 1 year. Finally, three studies identified a decrease in burnout levels 1 year after the intervention.^8,14,22^

Combined data analysis revealed a reduction in burnout levels in nine studies,^7-9,13-15,20-22^ whereas the rest of the studies did not identify any changes in burnout levels.^10-12,16-19^ No study identified increases in burnout levels at the end of the intervention.

## DISCUSSION

Most studies used the MBI to assess physician burnout. The MBI is used to assess burnout in practically any occupational setting. It consists of three dimensions: emotional exhaustion (exhaustion of emotional energy and fatigue), depersonalization (indifference toward or detachment from work), and work productivity (expectations of continuous work productivity).^23^ Using a single tool to assess burnout standardizes diagnostic criteria and, consequently, allows consistent observation and comparison of results.

The present literature review clearly shows that the interventions used to reduce physician burnout levels did not have any significant results. Of 16 studies, only nine had positive results (56.25%). Of these, three performed structural changes focused on the organization of the workplace, whereas the other six conducted individual-focused actions. Importantly, of the overall 16 studies, only five conducted changes focused on the workplace, corresponding to a burnout reduction success rate of 60%. The same success rate (60%) was observed for the remaining 10 studies, which performed individual-focused changes. Only one study conducted both interventions, but there were no positive results.

The reason why only a few studies implemented changes focused on the workplace is the difficulty in changing the organizational structure of medical practice. Reduction of working hours, changes in work shifts, and the improvement of team communication are some of the changes proposed in the studies, which require a great amount of time and money for the successful implementation of a new occupational structure.

Although interventions focused on the occupational environment are difficult to implement, improving communication between physicians and other members of the health care team has been shown to reduce burnout levels. Therefore, communication in the work environment should be improved, given that the health care team had an easy-to-understand and closed communication, which was implemented as a culture in the analyzed workplace.

On the other hand, interventions focused on changes in physicians’ shift schedules provided fewer positive results. Physicians continued to be subjected to an increased workload, a lack of control over their shift schedules, life and death decision-making regarding patients, and long working hours. When the method was successfully implemented, it ultimately resulted in reduced workload and working hours.

In most studies, individual-focused interventions were based on mindfulness, a methodology described by Kabat-Zinn^24^ aimed at keeping one’s attention focused on the present. Redirecting focus to what is happening in the present seems to be related to a reduction in self-perceived stress.^25^ Therefore, mindfulness-based programs are believed to be beneficial for individuals.

All studies that used mindfulness-based interventions successfully reduced burnout levels regardless of the follow-up period. This occurred because the method promotes self-awareness, elucidates the perception of psychological discomfort, and exposes barriers in the doctor-patient relationship, which results in better decision-making. The remaining studies, which used different approaches (such as self-care workshops, self-administered psychotherapeutic tools, and debriefing sessions), were not as successful as the studies that used mindfulness-based approaches. This is probably due to the short follow-up period in these studies.

The study using both interventions did not have significant results. The approach consisted of improving communication between members of the health care team - workplace-focused - and stress management sessions - individual-focused. Stress levels and professionals’ perception of productivity improved in a short time. However, results were not positive, given that the durations of the intervention and follow-up were not sufficient to influence burnout control.

The duration of follow-up varied between studies, and each study design was structured differently. This highlights how difficult it is for systematic reviews to conduct a more careful comparison of study conclusions.

Another limitation is that only a few studies had a follow-up period longer than 6 months. Although the studies with longer follow-up had positive results, we question whether the interventions are actually efficient in the long term.

## CONCLUSIONS

This literature review demonstrated the relevance of burnout syndrome among physicians, as well as the need to develop techniques for burnout reduction. In addition, besides benefiting physicians, interventions aimed at reducing burnout levels may also benefit those inserted in the occupational setting of health care professionals. Approaches focused both on the individual and the organization of the workplace are believed to be more effective at reducing burnout. However, further studies are needed to identify the best possible way to prevent the onset of burnout syndrome among physicians.
